# Increasing pulmonary artery visibility and diagnostic confidence with ultra-high resolution photon-counting detector CT pulmonary angiography

**DOI:** 10.1007/s00330-026-12588-3

**Published:** 2026-05-04

**Authors:** Pauline Pannenbecker, Camilla Rüth, Jan-Peter Grunz, Alena Kollmann, Philipp Gruschwitz, Julius Frederik Heidenreich, Andreas Steven Kunz, Thorsten Alexander Bley, Henner Huflage

**Affiliations:** 1 Department of Diagnostic and Interventional Radiology, University Hospital Würzburg, Würzburg, Germany; 2 Department of Radiology, University of Wisconsin—Madison, Madison, WI USA

**Keywords:** Computed tomography angiography, Pulmonary embolism, Radiation dosage

## Abstract

**Objectives:**

Photon-counting detector (PCD) CT allows for ultra-high-resolution (UHR) imaging of the pulmonary vasculature. The aim of this study was to assess the benefits of UHR-PCD-CT pulmonary angiography (CTPA) over dual-energy energy-integrating-detector (EID) CTPA.

**Materials and methods:**

Comparing UHR-PCD-CTPA (*n* = 76 at image quality index (IQ) 50, *n* = 76 at IQ25) and dual-energy EID-CTPA (*n* = 75) acquired between April and October 2024, a total of 227 examinations were analyzed in this retrospective single-center study after excluding 56 ineligible studies. Hounsfield unit measurements and subjective image quality ratings of three radiologists (e.g., peripheral pulmonary artery visibility and diagnostic confidence) were investigated.

**Results:**

Peripheral pulmonary artery visibility, overall image quality, and self-reported diagnostic confidence were higher in UHR-PCD-CTPA than in EID-CTPA (all *p* < 0.01), with no relevant differences between IQ25 and IQ50 scans (all *p* > 0.05). Diagnostic confidence was stronger in UHR-PCD-CTPA vs. EID-CTPA (the two highest confidence scores reported by readers after viewing CTPAs and iodine-maps in 92.1–100% of IQ50, 94.6–100% of IQ25 and 66.6–73.3% of EID cases). Median attenuation in the pulmonary trunk was higher in UHR-PCD-CTPA (IQ25: 623.3 HU, IQ50: 567.8 HU) compared with EID-CTPA (397.2 HU, all *p* < 0.01). CT dose index was highest for IQ50 UHR-PCD-CTPA (6.7 ± 2.4 mGy), compared to EID-CTPA (4.7 ± 2.7 mGy) and IQ25 UHR-PCD-CTPA (3.0 ± 1.0 mGy; all *p* < 0.01).

**Conclusion:**

Image quality of CTPA benefits greatly from employing the UHR-mode in PCD-CT. Compared to EID-CTPA, even UHR-PCD-CTPA scans with a markedly lower radiation dose facilitate superior intraluminal attenuation, peripheral pulmonary artery visibility, and diagnostic confidence.

**Key Points:**

***Question*** *With the introduction of a UHR scan option for PCD-CT of the pulmonary vasculature, the question arises whether patients benefit from this recent technological advancement.*

***Findings*** *UHR-PCD-CTPA offers improved image quality, increased pulmonary artery visibility, and higher diagnostic confidence, even with significant dose reduction compared to conventional EID-CTPA.*

***Clinical relevance*** *The advantages in subjective and objective image quality described for UHR-PCD-CTPA suggest potential for superior diagnostic performance in the work-up of pulmonary embolism.*

**Graphical Abstract:**

**Figure:**
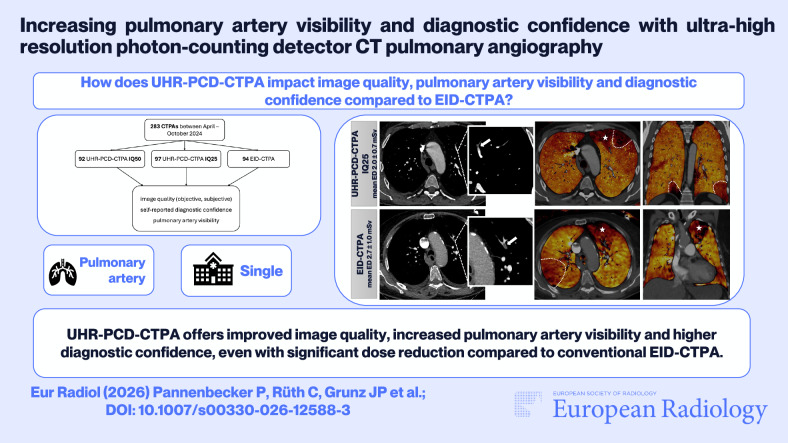

## Introduction

CT pulmonary angiography (CTPA) is the imaging modality of choice in the diagnosis of acute pulmonary embolism [1]. With the emergence of spectral CT, the quality of morphological images could be further improved by performing virtual monoenergetic reconstructions, increasing contrast. Moreover, iodine maps generated from spectral CT offer additional functional information, depicting pulmonary parenchymal perfusion at the time of image acquisition [2, 3]. These iodine maps have been shown to be beneficial in detecting small peripheral pulmonary emboli [4]. Up to 2020, most available technical approaches to spectral CT were based on energy-integrating detector (EID) technology. However, in 2020, a novel technical approach to spectral imaging became available with photon-counting detector (PCD) technology. First studies investigating the potential benefits of PCD-CTPA in the diagnosis of pulmonary embolism concluded that this detector technology enables scans with high-pitch settings, while also allowing for a substantial reduction of the required radiation dose and contrast medium quantities [5–8]. Employing semiconductor detector elements, PCD no longer requires separation of detector cells as opposed to conventional EID technology, which enables an ultra-high resolution (UHR) scan mode with a detector collimation of 120 × 0.2 mm. UHR-PCD-CT offers higher spatial resolution than EID-CT without dose penalty, while providing inherent spectral data in each scan, even in high-pitch settings. Making use of the small pixel effect, the UHR scan mode even allows for radiation dose reduction over standard-resolution PCD-CT (144 × 0.4 mm) [9, 10]. Previous investigations demonstrated image quality improvements with UHR-PCD-CT in the context of lung imaging, angiographies of the coronary arteries, the abdomen, and the lower extremities [11–18]. However, to date, no data are available concerning the feasibility, image quality, and diagnostic benefits of UHR-PCD-CTPA in the work-up of pulmonary embolism. Therefore, the aim of this study was to assess the impact of the UHR scan mode compared to state-of-the-art, dual-energy EID-CTPA.

## Material and methods

### Patient sample

This retrospective study received permission from the local institutional review board, which waived the need for additional written informed consent (20241031 01). A total of 283 adult patients undergoing CTPA to rule out pulmonary embolism in clinical routine between March and October 2024 were retrospectively analyzed. Of those, 94 had received dual-energy scans with an EID-CT scanner, while 92 and 97 consecutive individuals had received UHR-PCD-CTPA at an image quality index (IQ) level of 50 and 25, respectively. Exclusion criteria were deviations from scan protocols (e.g., varying amounts of contrast agent or flow rates), incomplete datasets or obesity with a body mass index ≥ 50 kg/m^2^, possibly limiting comparability.

### CT scan protocols

UHR-CTPA were performed on a first-generation, dual-source PCD-CT scanner (Naeotom Alpha.Peak, Siemens Healthineers). The dual-source setting allowed for image acquisition in flash mode with pitch set to 3.2 and a collimation of 120 × 0.2 mm. Images were acquired at a tube voltage of 140 kV at an IQ level of 50 or 25. The IQ index is a vendor-specific metric intended to provide a general indication of image quality; however, detailed information on its underlying calculation is not publicly available, and it therefore cannot be directly compared with standardized quantitative measures. UHR-PCD-CTPA were reconstructed in a medium soft vascular kernel (Bv48) at a virtual monoenergetic level of 55 keV with a slice thickness of 0.4 mm. For each scan, 40 mL of iodinated contrast medium (Imeron350, Bracco) was administered at a flow rate of 4 mL/s. EID-CTPA were acquired on a third-generation, dual-source scanner (Somatom Force, Siemens Healthineers) with an established scan protocol (i.e., dual-energy mode, pitch 0.55, tube voltage 90 kV/Sn 150 kV, reference mAs 70, collimation 2 × 96 × 0.6 mm). 50 mL of contrast medium (Imeron350, Bracco) was administered at a flow rate of 4 mL/s to ensure sufficient bolus length in lower pitch settings as required in dual-energy mode. Virtual blended images were reconstructed with a linear ratio of 0.6 and a soft vascular kernel (Bv36) at a slice thickness of 0.6 mm, representing the minimal slice thickness with this scan mode. Bolus-tracking was used in all protocols (100 HU trigger threshold, delay 5 s in UHR-PCD-CTPA; 120 HU trigger threshold, delay 7 s in EID-CTPA). Axial and coronal iodine maps were computed using dedicated software (Syngo.Via VB80D for EID-CTPA, VB10 for UHR-PCD-CTPA, Siemens Healthineers).

### Assessment of image quality

For objective image quality assessment, CT attenuation in Hounsfield units, signal-to-noise ratio (SNR), and contrast-to-noise ratio (CNR) were determined by one reader (CR) placing circular regions of interest within pulmonary vessels (i.e., main pulmonary artery, right lower lobe artery, and left upper lobe artery) and the erector spinae muscle. SNR and CNR were calculated as follows [19]:

$${{SNR}}=\frac{{{mean}}\,{{vessel}}\,{{attenuation}}\,[{{HU}}]}{{{{\rm{vessel}}}}\,{{noise}}\,\left({{standard}}\,{{deviation}}\,{{of}}\,{{vessel}}\,{{attenuation}}\right)\,[{{HU}}]}$$

$${{CNR}}=\frac{{{mean}}\,{{vessel}}\,{{attenuation}}\,\left[{{HU}}\right]-{{mean}}\,{{muscle}}\,{{attenuation}}\,[{{HU}}]}{{{vessel}}\,{{noise}}\,({{standard}}\,{{deviation}}\,{{of}}\,{{vessel}}\,{{attenuation}})\,[{{HU}}]}$$

Three radiologists with varying experience in cardiovascular imaging (reader 1 [R1], P.P., 4 years, reader 2 [R2], A.K., 1 year; reader 3 [R3], H.H., 10 years) performed randomized and blinded quality readings of every CTPA study. Categories of subjective image quality were: image quality ratings of CTPAs and iodine maps, assessment of peripheral pulmonary artery visibility and self-reported diagnostic confidence levels. For each category, scores attributed by every reader were compared.

For the evaluation of CTPA, rating scores were determined as follows: 5: excellent contrast, no motion or pulsation artifacts; 4: good contrast, minor motion or pulsation artifacts; 3: moderate contrast, moderate motion or pulsation artifacts; 2: poor contrast, major motion or pulsation artifacts; 1: insufficient contrast, drastic motion or pulsation artifacts. For the evaluation of iodine maps, the following rating scores were set: 5: excellent homogeneity, no artifacts; 4: good homogeneity, minor artifacts; 3: moderate homogeneity, moderate artifacts; 2: poor homogeneity, major artifacts; 1: insufficient homogeneity, drastic artifacts, iodine map non-diagnostic. Readers were asked to take inhomogeneity due to underlying pathologies (such as pulmonary emphysema or dystelectasis) into account when rating the image quality of iodine maps [20]. To quantify peripheral pulmonary artery visibility, the minimal distance from the last clearly visible pulmonary artery branch to the visceral pleura in the right lower lobe was measured. Additionally, readers were asked to estimate their subjective level of diagnostic confidence in reporting a study positive or negative for pulmonary embolism after reading CTPA only and after reading CTPA along with the respective iodine maps. In this context, the levels of diagnostic confidence were as follows: 5: very confident; 4: confident; 3: moderately confident; 2: hardly confident; 1: uncertain.

### Radiation dose

For each exam, radiation dose parameters (i.e., volume CT dose index (CTDI_vol_) and dose-length product (DLP)) were extracted from the patient report automatically generated by the scanner. The effective radiation dose was calculated using an established conversion factor for thorax CT imaging (i.e., $k=0.018\frac{{mSv}}{{mGy}\times {cm}}$) [21].

### Statistical analysis

Dedicated software was used for statistical analysis (SPSS version 29.0.1, IBM, Armonk, USA). Shapiro-Wilk tests were used to determine the normal distribution of continuous variables. In the case of normal distribution, items are reported as mean values ± standard deviation. Otherwise, we report median values with interquartile ranges. Ordinal-scaled data are presented as absolute and relative frequencies. Continuous items with normal distribution were compared among groups by means of one-way ANOVA with Tukey-HSD post-hoc tests. Non-parametric variables were compared by means of Kruskal-Wallis tests with Bonferroni correction for pairwise post-hoc testing. The intraclass correlation coefficient (ICC) was computed to assess interreader reliability based on absolute agreement in a two-way random effects model. Results were interpreted as suggested by Koo and Li, with coefficients > 0.9 indicating excellent, 0.75–0.9 good, 0.5–0.75 moderate, < 0.5 indicating poor interreader reliability [22]. *p*-values < 0.05 were considered statistically significant.

## Results

### Patient characteristics

**Fig. 1:**
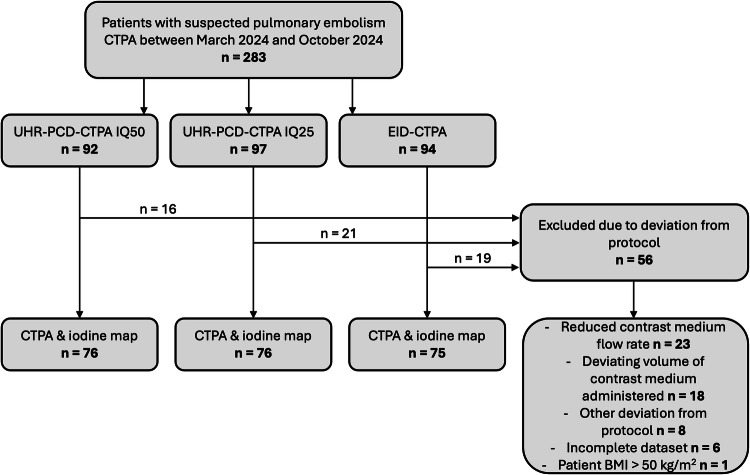
Study sample. “Other deviations from protocol” include cases in which patients had received contrast agent immediately before the diagnostic CTPA or in which regions of interest in monitoring were malpositioned, among others. BMI, body mass index; CTPA, CT pulmonary angiography; EID, energy-integrating detector; IQ, image quality index; PCD, photon-counting detector; UHR, ultra-high resolution

**Fig. 2:**
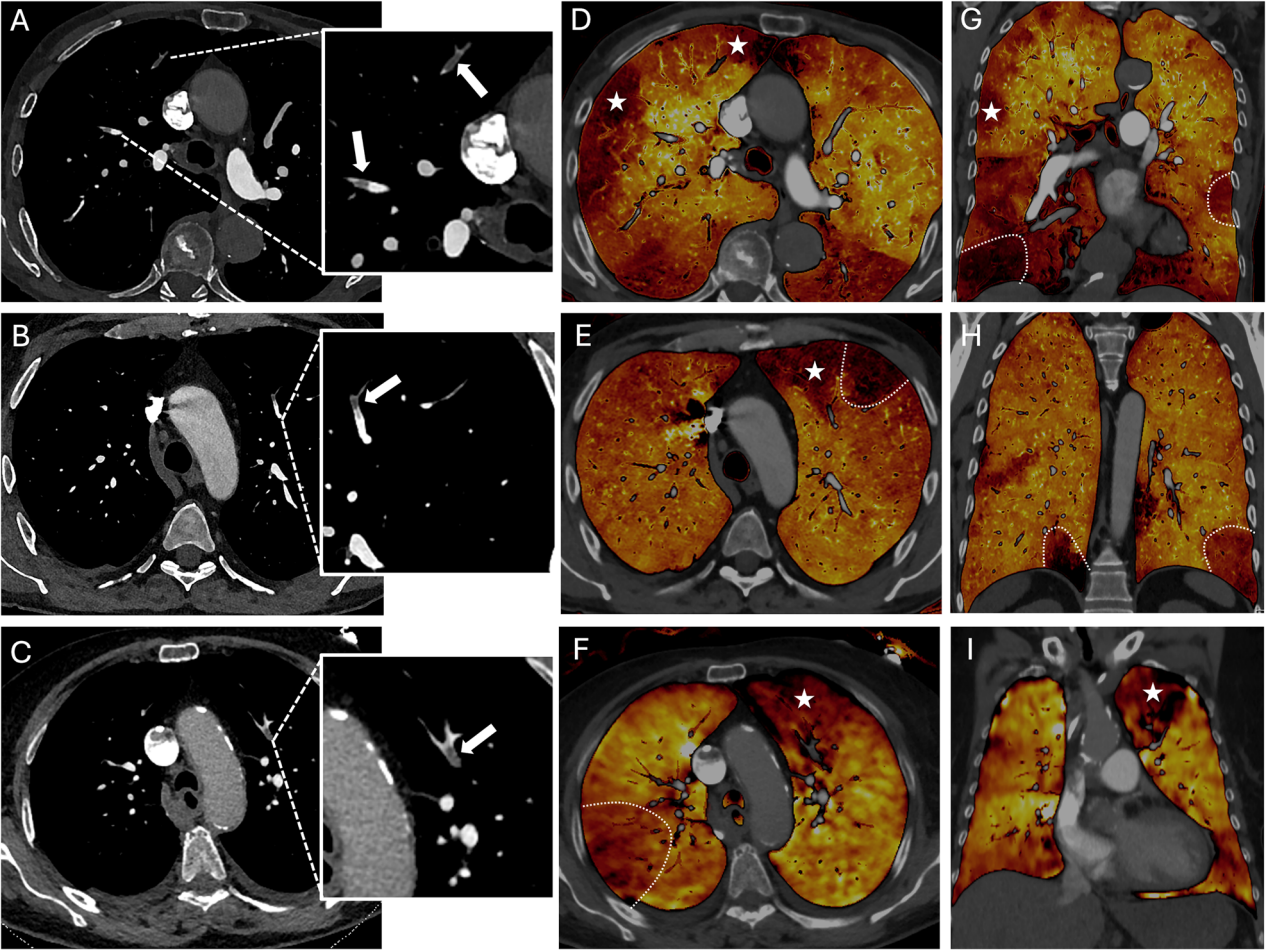
Multifocal peripheral pulmonary embolism. Axial images of UHR-PCD-CTPA at IQ50 (**A**) and IQ25 (**B**) vs. EID-CTPA (**C**) showing peripheral pulmonary embolism (magnified boxes, arrows) with consecutive perfusion deficits (asterisks) in the corresponding axial (**D**–**F**) and coronal (**G**–**I**) iodine maps. Perfusion deficits resulting from additional pulmonary emboli not depicted in the selected representative images are marked with dashed bows. Note the excellent spatial resolution of CTPA images and iodine maps with PCD in UHR mode, irrespective of IQ level. CTPA, CT pulmonary angiography; EID, energy-integrating detector; IQ, image quality index; PCD, photon-counting detector; UHR, ultra-high resolution

A total of 56 patients were excluded from the study sample (i.e., 16 in the PCD-CTPA IQ50 group, 21 in the PCD-CTPA IQ25 group, and 19 in the EID-CTPA group). The final patient sample comprised 227 patients (i.e., 76 UHR IQ50, 76 UHR IQ25, and 75 EID-CTPA). A flowchart visualizing the inclusion process and exclusions is given in Fig. 1. In the UHR-PCD-CTPA IQ50 group, 24 studies reported positive for pulmonary embolism (31.6%) vs. 20 in the UHR-PCD-CTPA IQ25 group (26.3%) and 16 in the EID-CTPA group (21.1%). Table 1 lists detailed demographic characteristics of each study group. Exemplary CTPA images of all groups positive for peripheral pulmonary embolism with resulting perfusion deficits in the corresponding iodine maps are shown in Fig. 2.

**Table 1:** Study sample

Patient characteristics	UHR-PCD-CTPA IQ50	UHR-PCD-CTPA IQ25	EID-CTPA	*p*-value
				ANOVA
Men	34 (44.7%)	45 (59.2%)	31 (41.3%)	
Women	42 (55.3%)	31 (40.8%)	44 (58.7%)	
Age (mean ± standard deviation) (years)	64.1 ( ± 14.8)	66.6 ( ± 16.3)	65.5 ( ± 15.0)	0.6
Body mass index (mean ± standard deviation) (kg/m^2^)	27.4 ( ± 6.0)	27.3 ( ± 5.2%)	26.2 ( ± 4.6)	0.31
CTPA positive for pulmonary embolism	24 (31.6%)	20 (26.3%)	16 (21.3%)	
	9 central 15 peripheral	3 central 17 peripheral	5 central 11 peripheral	
CTPA negative for pulmonary embolism	52 (68.4%)	56 (73.7%)	59 (78.7%)	
Perfusion deficits in iodine maps	26 (34.2%)	18 (23.7%)	16 (21.3%)	
No perfusion deficits in iodine maps	50 (65.8%)	58 (76.3%)	59 (78.7%)	

Unless declared otherwise, results are given as absolute numbers with percentages in parentheses

*CTPA* CT pulmonary angiography, *EID* energy-integrating detector, *IQ* image quality index, *PCD* photon-counting detector, *UHR* ultra-high-resolution

### Image quality

#### Objective measures of image quality

Mean attenuation within the pulmonary vasculature was higher in both UHR-PCD-CTPA groups compared to the EID-CTPA group (all *p* < 0.01), e.g., 567.8 HU (IQ50) and 623.3 HU (IQ25) vs. 397.2 HU (EID-CTPA) within the pulmonary trunk (all *p* < 0.01). No relevant difference in CT attenuation was observed between the IQ50 and IQ25 group (all *p* ≥ 0.05). Furthermore, no statistically significant difference in SNR and CNR was ascertained between the three groups (all *p* ≥ 0.05). For detailed objective image quality parameters, see Table 2.

**Table 2:** Objective image quality parameters

				Post-hoc test		
	UHR-PCD-CTPA IQ50	UHR-PCD-CTPA IQ25	EID-CTPA	*p*-value	*p*-value	*p*-value
				IQ50 vs. IQ25	IQ50 vs. EID	IQ25vs. EID
Pulmonary trunk						
Attenuation (HU)	567.8 (470.7–713.5)	623.3 (524.9–753.7)	397.2 (304.5–481.6)	0.68	< 0.01	< 0.01
Noise (HU)	36.7 (33.9–40.0)	45.9 (40.6–47.7)	24.2 (22.9–27.3)	< 0.01	< 0.01	< 0.01
SNR	14.9 (12.5–18.4)	13.8 (12.0–17.0)	15.4 (12.3–18.3)	0.25	1.0	0.22
CNR	13.4 (10.9–17.1)	12.7 (10.8–15.6)	13.5 (10.1–16.2)	0.51	1.0	1.0
Right lower lobe artery						
Attenuation (HU)	593.2 (468.0–732.6)	630.7 (517.3–718.4)	380.1 (289.8–466.1)	1.0	< 0.01	< 0.01
Noise (HU)	33.3 (30.1–38.8)	39.3 (35.9–43.0)	22.8 (19.1–26.2)	< 0.01	< 0.01	< 0.01
SNR	17.7 (14.1–20.8)	16.0 (12.5–18.9)	15.4 (12.5–21.3)	0.17	0.72	1.0
CNR	16.4 (12.1–19.0)	14.6 (11.0–17.4)	13.1 (10.1–18.8)	0.31	0.18	1.0
Left upper lobe artery						
Attenuation (HU)	596.2 (489.6–731.5)	631.2 (509.4–706.4)	349.1 (280.3–438.2)	1.0	< 0.01	< 0.01
Noise (HU)	31.4 (28.7–37.3)	37.4 (33.0–42.2)	21.3 (18.5–26.4)	0.01	< 0.01	< 0.01
SNR	17.6 (13.1–23.7)	16.5 (12.7–19.7)	15.3 (12.1–20.9)	1.0	0.44	1.0
CNR	15.8 (11.5–21.7)	15.1 (11.5–17.9)	12.4 (9.6–18.7)	1.0	0.09	0.53

Results are given as median values with interquartile ranges in parentheses

*CNR* contrast-to-noise ratio, *CTPA* CT pulmonary angiography, *EID* energy-integrating detector, *IQ* image quality index, *PCD* photon-counting detector, *SNR* signal-to-noise ratio, *UHR* ultra-high-resolution

#### Subjective image quality

Subjective CTPA image quality was rated higher in both UHR-PCD groups (excellent or good) than in the EID group (moderate or good; all *p* < 0.01), while image quality ratings did not differ in a statistically significant way between the IQ50 and IQ25 samples (all *p* ≥ 0.05). Interreader reliability was good with an ICC of 0.830 (0.787–0.866; *p* < 0.01).

Iodine maps from UHR-PCD-CTPA datasets were rated as superior (excellent or good) to those from EID-CTPA scans (moderate or good; all *p* < 0.01) by all readers, while image quality ratings were similar between both UHR-PCD-CTPA groups (all *p* ≥ 0.05). Interreader reliability was good, as indicated by an ICC of 0.753 (0.688–0.866; *p* < 0.01). In Table 3, median image quality scores of CTPAs and iodine maps with post-hoc tests are documented.

**Table 3:** Subjective image quality of CT pulmonary angiographies and iodine maps

CTPA				Post-hoc test		
	UHR-PCD-CTPA IQ50	UHR-PCD-CTPA IQ25	EID-CTPA	*p*-value	*p*-value	*p*-value
				IQ50 vs. IQ25	IQ50 vs. EID	IQ25vs. EID
Reader 1	5 (4–5)	5 (5–5)	3 (3–4)	1.0	< 0.01	< 0.01
Reader 2	4 (4–5)	5 (4–5)	4 (3–4)	1.0	< 0.01	< 0.01
Reader 3	5 (4–5)	4 (4–5)	4 (3–4)	0.76	< 0.01	< 0.01
ICC (95% CI, *p*-value)	0.830 (0.787–0.866; *p* < 0.01)					

Iodine map				Post-hoc test		
	UHR-PCD-CTPA IQ50	UHR-PCD-CTPA IQ25	EID-CTPA	*p*-value	*p*-value	*p-*value
				IQ50 vs. IQ25	IQ50 vs. EID	IQ25vs. EID
Reader 1	5 (5–5)	5 (4–5)	3 (3–4)	0.2	< 0.01	< 0.01
Reader 2	5 (4–5)	5 (4–5)	4 (4–4)	0.8	< 0.01	< 0.01
Reader 3	4 (4–5)	4 (4–5)	4 (3–4)	0.2	< 0.01	< 0.01
ICC (95% CI, *p-*value)	0.753 (0.688–0.806; *p* < 0.01)					

Results are given as median values with interquartile ranges in parentheses

Rating scores: 5 = excellent contrast, no motion or pulsation artifacts; 4 = good contrast, minor motion or pulsation artifacts; 3 = moderate contrast, moderate motion or pulsation artifacts; 2 =  poor contrast, major motion or pulsation artifacts; 1 = insufficient contrast, drastic motion or pulsation artifacts

*CI* confidence interval, *CTPA* CT pulmonary angiography, *EID* energy-integrating detector, *ICC* intraclass correlation coefficient, *IQ* image quality index, *PCD* photon-counting detector, *UHR* ultra-high-resolution

#### Peripheral pulmonary artery visibility

**Fig. 3:**
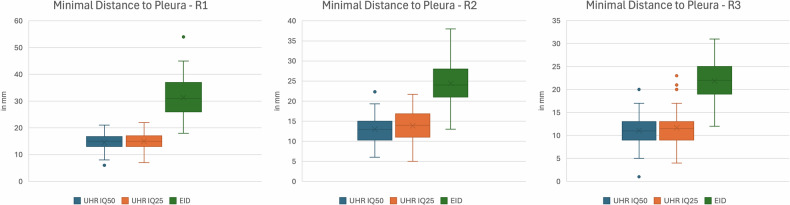
Lower distance from the peripheral pulmonary artery to pleura measured with ultra-high resolution photon-counting CT. Boxplots demonstrate median values of minimal distance from the last clearly discernible peripheral pulmonary artery branch to the visceral pleura as measured by readers 1-3 (R1-3). Irrespective of dose level, readers consistently measured a lower distance in the UHR-PCD-CPTA groups than in the EID CTPA group. CTPA, CT pulmonary angiography; EID, energy-integrating detector; IQ, image quality index; PCD, photon-counting detector; UH, ultra-high resolution

**Fig. 4:**
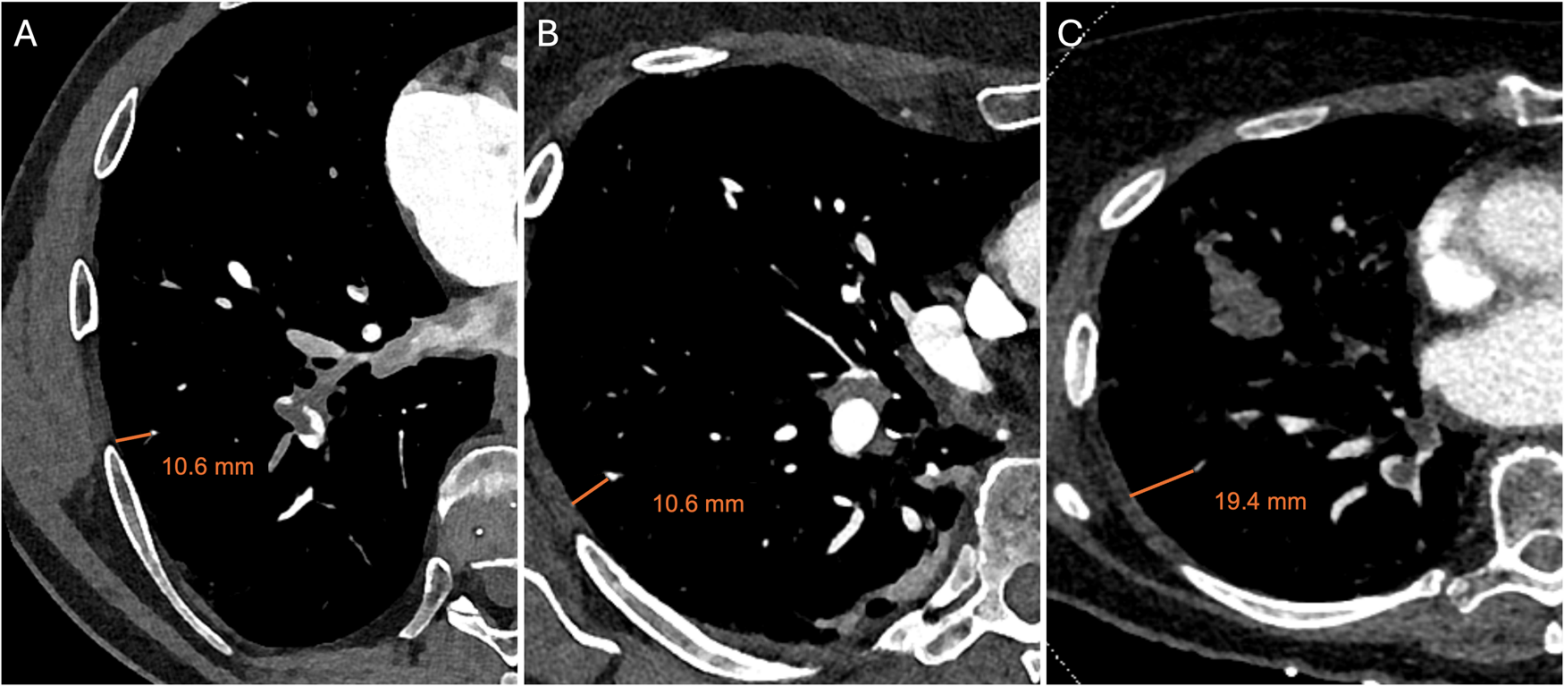
Increased peripheral pulmonary artery visibility with ultra-high-resolution photon-counting CT. UHR-PCD-CTPA at IQ50 (**A**) and IQ25 (**B**) vs. EID-CTPA (**C**). Representative measurements of the minimal distance of the last discernible pulmonary artery branch to the pleura are shown (orange line, in mm). CTPA, CT pulmonary angiography; EID, energy-integrating detector; IQ, image quality index; PCD, photon-counting detector; UHR, ultra-high resolution

Visibility of peripheral pulmonary vessels was deemed higher in both PCD-CTPA samples vs. EID-CTPA with a median minimal distance to the pleura of 15.0 mm (13.0–16.3 mm) measured by R1, 13.0 mm (10.5–15.0 mm) by R2, and 11.0 mm (9.0–13.0 mm) by R3 in the IQ50 group, as well as 15.0 mm (13.0–17.0 mm) measured by R1, 14.0 mm (11.1–16.6 mm) by R2, and 11.5 mm (9.0–13.0 mm) by R3 in the IQ25 group. Minimal distance to pleura was highest in the EID-CTPA group with a median of 31 mm (26.5–36.5) measured by R1, 24 mm (21.0–28.0 mm) by R2 and 22 mm (19.0–25.0 mm) by R3. Post-hoc tests showed no significant differences between IQ50 and IQ25 measurements for all readers (*p* = 1.0 for R1 and R3, *p* = 0.89 for R2), while EID-CTPA measurements were significantly higher than measurements in both UHR-PCD-CTPA groups (all *p* < 0.01, see Fig. 3). Interreader reliability for minimal pleura distance was good, indicated by an ICC of 0.826 (95% confidence interval 0.692–0.892; *p* < 0.01). In Fig. 4, representative measurements are depicted for all three groups.

#### Diagnostic confidence

All readers reported higher diagnostic confidence when reading UHR-PCD-CTPA compared with EID-CTPA: In the IQ50 cohort, two readers felt very confident, and one felt confident in confirming or excluding pulmonary embolism, while all readers felt confident in their diagnosis in the IQ25 group. In the EID-CTPA group, confidence was lower, with two readers feeling confident and one reader moderately confident in their diagnosis. Interreader reliability was good, indicated by an ICC of 0.785 (0.731–0.830; *p* < 0.01).

**Fig. 5:**
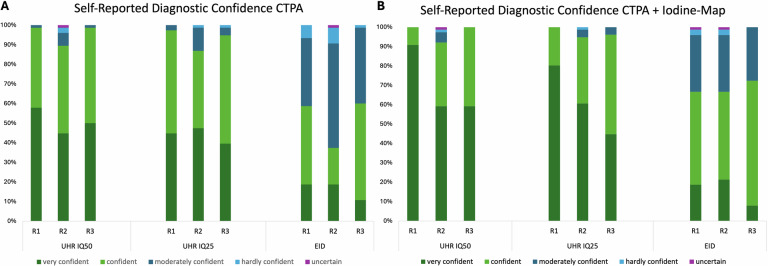
Higher self-reported diagnostic confidence with ultra-high resolution photon-counting CT. Stacked bar charts demonstrating self-reported diagnostic confidence levels of readers 1-3 (R1-3). Readers reported higher confidence levels in confirming or excluding pulmonary embolism for UHR-PCD-CTPAs at IQ50 and IQ25 than for EID-CTPAs, after reading CTPA alone (**A**) as well as CTPA and iodine-maps combined (**B**, the two highest confidence scores reported by readers after viewing CTPAs and iodine-maps in 92.1–100% of IQ50, 94.6–100% of IQ25 and 66.6–73.3% of EID cases). CTPA, CT pulmonary angiography; EID, energy-integrating detector; IQ, image quality index; PCD, photon-counting detector; UHR, ultra-high resolution

**Fig. 6:**
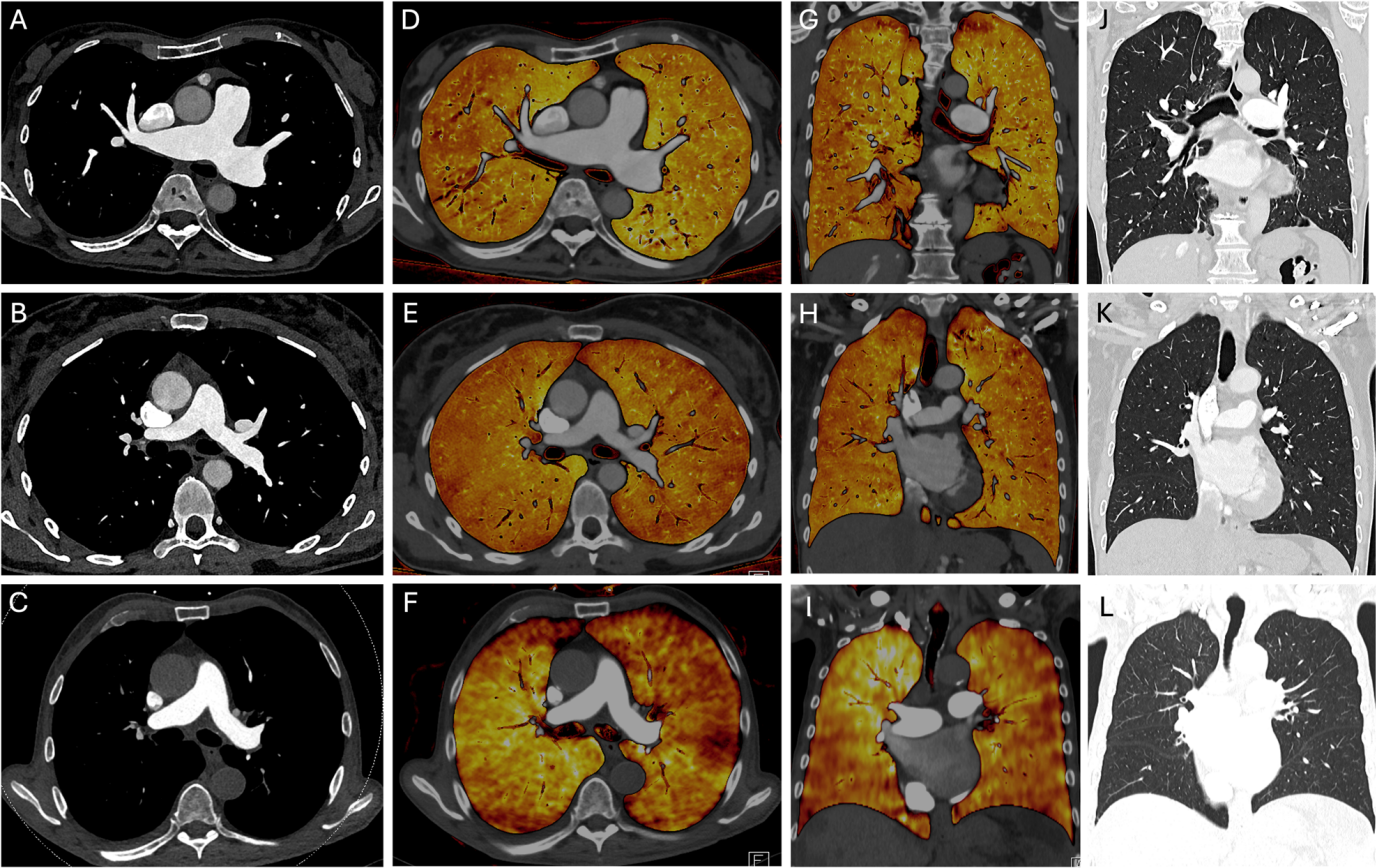
Exclusion of pulmonary embolism. Axial images of UHR-PCD-CTPA at IQ50 (**A**) and IQ25 (**B**) vs. EID-CTPA (**C**) with corresponding axial (**D**–**F**) and coronal (**G**–**I**) iodine maps, as well as coronal slices of the corresponding lung window (**J**–**L**). Note the excellent homogeneity of iodine maps of PCD-CTPA in UHR mode, irrespective of IQ level, while EID-CTPA iodine maps show some inhomogeneous spots that are not explained by underlying parenchymal pathologies like emphysema (see corresponding coronal lung window, **L**). CTPA, CT pulmonary angiography; EID, energy-integrating detector; IQ, image quality index; PCD, photon-counting detector; UHR, ultra-high resolution

When reading CTPA and iodine maps combined, all readers felt very confident in the UHR-PCD-CTPA IQ50 cohort, while two readers felt very confident and one felt confident in the UHR-PCD-CTPA IQ25 group, and all readers reporting an EID-based study felt confident in their diagnosis. Interreader reliability was good with an ICC of 0.788 (0.729–0.834; *p* < 0.01). The stacked bar chart in Fig. 5 gives a detailed comparison of readers’ self-reported diagnostic confidence when reading CTPA alone and CTPA and iodine maps together, median confidence scores are listed in Table 4. A subgroup analysis of all studies negative for pulmonary embolism showed similar results in self-reported diagnostic confidence: self-reported confidence scores were higher in the UHR-PCD-CTPA groups than in the EID-CTPA group (all *p* < 0.01), while no statistically significant difference was ascertained between IQ50 and IQ25 (all *p* ≥ 0.05, Fig. 6).

**Table 4:** Self-reported diagnostic confidence

CTPA alone				Post-hoc test		
	UHR-PCD-CTPA IQ50	UHR-PCD-CTPA IQ25	EID-CTPA	*p*-value	*p*-value	*p*-value
				IQ50 vs. IQ25	IQ50 vs. EID	IQ25vs. EID
Reader 1	5 (4–5)	4 (4–5)	4 (3–4)	0.5	< 0.01	< 0.01
Reader 2	4 (4–5)	4 (4–5)	3 (3–4)	1.0	< 0.01	< 0.01
Reader 3	5 (4–5)	4 (4–5)	4 (3–4)	0.51	< 0.01	< 0.01
ICC (95% CI, *p*-value)	0.785 (0.731–0.830; *p* < 0.01)					
**CTPA + Iodine map**				**Post-hoc test**		
	**UHR-PCD-CTPA IQ50**	**UHR-PCD-CTPA IQ25**	**EID-CTPA**	***p*-value**	***p*-value**	***p*-value**
				IQ50 vs. IQ25	IQ50 vs. EID	IQ25vs. EID
Reader 1	5 (4–5)	5 (5–5)	4 (3–4)	0.73	< 0.01	< 0.01
Reader 2	5 (5–5)	5 (4–5)	4 (3–4)	1.0	< 0.01	< 0.01
Reader 3	5 (5–5)	4 (4–5)	4 (3–4)	0.2	< 0.01	< 0.01
ICC (95% CI, *p*-value)	0.788 (0.729–0.834; *p* < 0.01)					

Results are given as median values with interquartile ranges in parentheses

*CI* confidence interval, *CTPA* CT pulmonary angiography, *EID* energy-integrating detector, *ICC* intraclass correlation coefficient, *IQ* image quality index, *PCD* photon-counting detector, *UHR* ultra-high-resolution

### Radiation dose

Mean CTDI_vol_ was highest in the UHR-PCD-CTPA IQ50 group (6.7 ± 2.4 mGy) and lowest in the IQ25 group (3.0 ± 1.0 mGy), whereas the mean CTDI_vol_ administered to patients in the EID-CTPA group was between the two UHR-PCD-CTPA groups (4.7 ± 2.7 mGy, all *p* < 0.01). Correspondingly, the dose-length product and effective radiation exposure were also higher in PCD-CTPA IQ50 and lower in IQ25 compared with EID-CTPA (all *p* < 0.01). Detailed radiation dose results are given in Table 5.

**Table 5:** Radiation dose

Radiation dose parameters	UHR-PCD-CTPA IQ50	UHR-PCD-CTPA IQ25	EID-CTPA	*p*-value
Volume CT dose index (mGy)	6.7 ± 2.4	3.4 ± 1.0	4.7 ± 2.7	< 0.01
Dose-length product (mGy × cm)	222.2 ± 77.8	110.5 ± 37.5	148.3 ± 57.8	< 0.01
Effective dose (mSv)	4.0 ± 1.4	2.0 ± 0.7	2.7 ± 1.0	< 0.01

Results are given as mean ± standard deviation. To improve readability, *p*-values were summarized, and post-hoc tests ascertained significance in all comparisons (all *p* < 0.01)

*CTPA* CT pulmonary angiography, *EID* energy-integrating detector, *IQ* image quality index, *PCD* photon-counting detector, *UHR* ultra-high-resolution

## Discussion

This study evaluated the image quality, pulmonary artery visibility, and diagnostic confidence of CTPAs acquired with the UHR scan mode of a first-generation dual-source PCD-CT scanner compared to state-of the art dual-energy EID-CT. The results show that UHR high-pitch CTPAs with PCD technology allow for superior subjective quality of morphological CT images and iodine maps, resulting in increased visibility of the peripheral pulmonary arteries and ultimately higher diagnostic confidence compared with established EID-CT scans.

The impact of the ultra-high resolution scan mode on image quality and radiation dose has been investigated in several previous studies. Huflage et al and Fix Martinez et al observed that the use of a smaller detector element size in this scan mode facilitates an increase in CNR [9]. This so-called small pixel effect can be used for substantial radiation dose reduction in imaging of the lung and lumbar spine, among others [10, 23]. The results of this study are in line with the observations described in the literature: we demonstrate that CTPA in UHR scan mode with PCD-CT is feasible and offers relevant benefits over conventional EID-CTPA, namely higher CT attenuation within the pulmonary arteries and higher self-reported diagnostic confidence for both UHR-PCD-CTPA groups over EID-CTPA, as well as the potential of substantial radiation dose reduction. Furthermore, median CNR within the lobar pulmonary arteries leaned higher in the UHR-PCD groups than in the EID-CTPA group (without reaching statistical significance in this investigation), likely due to the observed increased CT attenuation. Image noise was significantly higher in UHR-PCD, likely due to a smaller slice thickness. However, adopting the scan parameters from previous investigations on PCD-CTPA in standard-resolution mode, e.g., an IQ level of 50 [24], UHR-PCD-CTPA examinations were associated with an increased CTDI_vol_ and effective dose of 6.7 ± 2.4 mGy and 4.0 ± 1.4 mSv, respectively, compared with standard EID-CTPA. To account for this fact, a second PCD-based protocol in UHR mode with an IQ level of 25 was included in the analysis, allowing for a radiation dose reduction of nearly 50% over EID-CTPA and reaching similar radiation dose levels as previously described in the literature [5, 25]. This finding suggests that UHR protocols cannot be simply matched to existing standard-resolution settings but require deliberate adjustments. Of note, even in low-dose scenarios (i.e., IQ25), UHR-PCD-CTPA delivered superior pulmonary artery visibility and image quality compared to EID-CTPA, probably resulting in higher self-reported diagnostic confidence.

A key result of this study is that the use of UHR-PCD for CTPA increases peripheral pulmonary artery visibility. While this was not an objective of this study, it can be speculated that the increased visibility translates into an easier detection of small peripheral pulmonary embolisms compared to standard EID-CTPA. Notably, this assumption is based on the reader assessment within this investigation, and a dedicated analysis of diagnostic accuracy was not in the scope of the study. Determining the appropriate treatment for patients with subsegmental pulmonary embolism remains controversial in clinical routine, with several studies suggesting a higher recurrence rate in patients with subsegmental embolisms compared to more central embolisms [26–29]. With that in mind, the impact of UHR-PCD-CTPA on diagnostic performance and clinical management of subsegmental pulmonary embolisms needs to be evaluated in future studies.

Several limitations of this study need to be addressed. First, this was a retrospective, single-center study. Despite this drawback, a patient sample of 227 individuals in total can be considered sufficient to provide the required statistical power for the targeted investigation. Second, various post-processing differences between EID-CT and PCD-CT, e.g., slice thickness of 0.6 mm vs. 0.4 mm and the use of a Bv36 kernel vs. a sharper Bv48 kernel, may have influenced the image quality in general and the image noise level in particular. Even with settings prioritizing spatial resolution over CNR, UHR-PCD-CTPA surpassed EID-CTPA in all subjective categories. Third, subjective image quality was assessed with one rating score per study, combining contrast and motion artifacts. This may limit sensitivity to subtle group differences, particularly since the lower pitch in the EID-CTPA group can increase such artifacts. Higher CT attenuation values within the UHR-PCD groups than in the EID group may have further influenced subjective image quality ratings. Additionally, previous studies on PCD-CT observed higher attenuation values in female subjects [30]. A dedicated subgroup analysis of attenuation between sexes was not within the scope of this investigation, but needs to be considered when interpreting the results. Moreover, the benefits of PCD-CTPA in UHR mode were not compared to PCD-CTPA in standard resolution mode. This aspect should be addressed in a future investigation. Finally, the influence of different convolution kernels on the reconstruction of UHR-PCD-CTPA datasets was not assessed. A recent study by Stammen et al analyzed CTPA images reconstructed with a lower vascular kernel (Bv40) than the one chosen in this study (Bv48) [31]. It may be noted that previous studies on other body parts suggest that the choice of reconstruction kernel can further improve image quality of UHR-PCD-CT scans [12, 32–34].

In conclusion, the image quality of CTPA benefits greatly from the use of high-pitch PCD-CT in UHR mode. Compared to conventional EID-CTPA, even UHR-PCD-CTPA scans with a markedly lower radiation dose allow for superior intraluminal attenuation, peripheral pulmonary artery visibility, and ultimately diagnostic confidence.

## Abbreviations

CTDI_vol_: Volume computed tomography dose index
CTPA: Computed tomography pulmonary angiography
EID: Energy-integrating detector
ICC: Intraclass correlation coefficient
IQ: Image quality index (vendor-specific dimensionless index)
PCD: Photon-counting detector
UHR: Ultra-high resolution
